# HPV monitoring in kidney transplanted patients

**DOI:** 10.1186/1750-9378-7-S1-P15

**Published:** 2012-04-19

**Authors:** Franco M Buonaguro, MariaLina Tornesello, Luigi Buonaguro

**Affiliations:** 1 Unit of Molecular Biology and Viral Oncogenesis & AIDS Reference Center, Istituto Nazionale Tumori "Fondazione Giovanni Pascale", Naples, Italy

## Background

Renal allograft recipients, as for HIV/AIDS patients, have a well-documented increased incidence of human papillomavirus (HPV)-related malignancies and preventive strategies should be specifically implemented. While in females the use of the Papanicolau test and HPV detection assay are used currently as a screening test for cervical cancer, no diagnostic procedures have been implemented to monitor HPV infection in males. The aim of this study was to test for HPV infection and to determine the spectrum of viral genotypes in urine samples of men with renal transplants.

## Material and methods

The study included 103 patients who underwent kidney transplantation between 1999 and 2008. HPV sequences were detected by nested PCR, using the broad-spectrum consensus- primer pairs MY09/MY11 and the new MGP system, and characterized by nucleotide sequence analysis.

## Results

Overall, 49 (47.5%) samples were found positive for HPV sequences and the most common genotypes were HPV 16 (51.0%) and HPV 54 (10.2%) followed by HPV6, 53, 56, 58, 66, 11, 12, 20, 45, 62, and 71, in descending order of prevalence (Table 1). The majority of HPV 16 isolates were classified as European and only two as African-1 variant on the basis of nucleotide signature present within the MGP L1 region.

**Table 1:** Prevalence of HPV genotypes in renal transplant patients

HPV genotype^a^	HPV positive, n (%)
HPV positive	49 (47.5)
HPV negative	54 (52.4)
	
Single infections	
16	25 (51.0)
56	2 (4.0)
58	1 (2.0)
54	5 (10.2)
6	2 (4.0)
11	1 (2.0)
12	1 (2.0)
20	1 (2.0)
53	1 (2.0)
62	1 (2.0)
66	1 (2.0)
71	1 (2.0)
Total single infections	42 (85.7)
	
Total multiple infections	4 (8.2)
	
Undetermined	3 (6.1)

^a^Gray shadow indicates HPV genotypes defined by IARC working group as class I carcinogens for humans [Bouvard et al., Lancet Oncol 10:321–322, 2009]

## Conclusion

The high prevalence of HPV 16 among renal allograft recipients suggests that an HPV-16-based preventive or therapeutic vaccine may be effective for prevention or treatment of HPV-related neoplasia in this group of immune compromised patients.
